# PROTOCOL: Effectiveness of home‐based interventions to prevent child neglect: A systematic review

**DOI:** 10.1002/cl2.1373

**Published:** 2023-12-06

**Authors:** Yanfei Li, Rui Li, Meixuan Li, Zhitong Bing, Xiuxia Li, Kehu Yang

**Affiliations:** ^1^ Centre for Evidence‐Based Medicine, School of Basic Medical Sciences Lanzhou University Lanzhou China; ^2^ Centre for Evidence‐Based Social Science, School of Public Health Lanzhou University Lanzhou China; ^3^ China National Health Development Research Center Beijing China; ^4^ Institute of ModernPhysics Chinese Academy of Sciences Lanzhou China

## Abstract

This is the protocol for a Campbell systematic review. The objectives are as follows. The objectives of the present study are to answer the following questions: (1) What types of home‐based interventions are currently being studied to prevent child neglect? (2) How effective are the different home‐based interventions for preventing child neglect? (3) What are the causes of heterogeneity among included studies and their impact on study effects?

## 1 BACKGROUND

### 1.1 Description of the condition

Neglect, which is the most common type of child maltreatment, is most frequently associated with child fatality (HHS, 2022). According to the World Health Organization (WHO) global status report on preventing violence against children 2020 and responding to child maltreatment: a clinical handbook for health professionals, 16% of children have been neglected (WHO, 2022). Median rates of neglect were highest in Africa (girls 42%, boys 39%) and South America (girls 55%, boys 57%). By contrast, median rates of neglect differed between the sexes in North America (girls 41%, boys 17%) but were similar in Asia (girls 26%, boys 24%) (WHO, 2020). Additionally, in the United States, according to the U.S. Department of Health & Human Services Child Maltreatment 2021 (HHS, 2023), there are 600,000 victims of maltreatment in 2021, and three‐quarters (76.0%) of victims are categorized as neglect. A nationally estimated 1820 children died as a result of abuse and neglect, with 77.7% suffering neglect. The Consultation on Child Abuse Prevention has defined child neglect as “In the context of resources reasonably available, family or caretaker's failure to provide for the development of the child in all spheres, including health, education, emotional development, nutrition, shelter, and safe living conditions, and cause or have a high probability of causing harm to the child's health or physical, mental, spiritual, moral or social development” (WHO, 1999). In general, child neglect is a heterogeneous construct encompassing rather dissimilar negative child experiences, and the several basic categories of child neglect include educational neglect, emotional neglect, inadequate supervision, medical neglect, and physical neglect (Child Welfare Information Gateway, 2018).

Multiple risk factors of child neglect have been identified, of which family‐related factors are the most prominent. These factors include having a history of antisocial behavior/criminal offenses, having a history of mental/psychiatric problems, having a history of mental or physical problems, and having a low educational level among parents (Mulder, 2018). The mental, physical, and/or behavioral problems of children were also found to increase the risk of child neglect. Meanwhile, various studies have shown a range of possible adverse consequences of child neglect. Specifically, research indicates that child neglect might lead to an additional 3‐month syntactic delay, subsequently impacting cognitive function (Kobulsky, 2022; Naughton, 2013; Spratt, 2012). Across various studies, children subjected to neglect face a significantly higher risk (two to three times) for depressive, anxiety, eating, and behavioral/conduct disorders, with a notable dose–response relationship for depressive disorders (Norman, 2012). Furthermore, correlation analysis research suggests that child neglect is associated with diminished task involvement, lower creativity, and difficulty discerning emotional expressions in peers. Additionally, it is linked to lower overall intelligence and self‐esteem scores (Kobulsky, 2022; Naughton, 2013). Moreover, children subjected to neglect experience increased disciplinary problems, suspensions, grade retention, and special educational needs, accompanied by deficits in emotional knowledge (Maguire, 2015). The health consequences of child neglect extend to changes in brain maturation, dental caries, injuries, and, in severe cases, death (Avdibegović, 2020; Brandon, 2014; Fisher‐Owens, 2017).

While it remains important to quantify and understand the physical and behavioral health impacts of child abuse and neglect, adequate literature has established foundational justification to support prevention efforts (Ogle, 2022; Welch, 2013). For example, certain epidemiological studies on the association of adverse childhood experiences (ACE) with physical health have demonstrated a lower impact of neglected early ACEs versus chronic ACEs, emphasizing the potential of meaningful intervention to reduce the risk trajectory (Chaiyachati, 2021; Thompson, 2015). The prevention of child abuse and neglect has become a public health priority, given its now well documented impact on the health and wellbeing of a developing child (Berger, 2015; Centers for Disease Control [CDC], 2016; Guterman, 2013). Over the past few decades, the evidence base that supports preventive interventions has increased significantly, with promising findings emerging from various preventive strategies, particularly home‐based interventions (Ash, 2017; Guastaferro, 2022). The United Nations International Children's Emergency Fund (UNICEF) supplemented in‐person group and home‐based programs with innovative remote and digital delivery modalities to enhance prevention of violence, exploitation, abuse, and neglect (UNICEF, 2023). The CDC developed a technical package for preventing child abuse and neglect. The package includes the following five strategies for prevention: strengthen economics, change social norms to support parents and positive parenting, provide quality care and education, enhance parenting skills to promote healthy child development, and intervene to lessen harms and prevent future risk; home visiting programs is one of the key ways to implement these strategies (CDC, 2016). The 2018 Family First Prevention Services Act requires the states in the US to develop, operate, expand, and enhance community‐based, prevention‐focused programs and activities designed to strengthen and support families to prevent child abuse and neglect (HHS, 2018). In 2018, the US Preventive Services Task Force (USPSTF) performed an evidence review of primary care interventions to prevent child maltreatment and found that the most common research included in the evidence review was the studies on home visitation‐based interventions (Curry, 2018).

### 1.2 Description of the intervention

In general, the goals of home‐based intervention include providing parents with information, emotional support, access to other services, and direct instruction on parenting practices (although programs vary in how they achieve these goals as well as in the relative importance placed on the goals) (Dongmo, 2021; Howard, 2009; Pamungkas, 2019; Welsh, 2011; Williams, 1995).

Home‐based intervention is an increasingly popular method for delivering services to families. In particular, for high‐risk families with infants and young children, providing services within the context of the family's home appears a useful and effective strategy in preventing or reducing the incidence of childhood diseases (Berger, 2015; Curry, 2018; Dongmo, 2021; Haines, 2018; Mirotta, 2018; Pamungkas, 2019; Taverno, 2017). Many different programs or models of home‐based interventions exist to prevent or reduce the incidence of child neglect. For example, The Australian Institute of Family Studies performed comparative analysis using 10 different home visiting programs for preventing child abuse or neglect. These programs aimed to improve parenting competence and enhance child development. Moreover, they provided parents with education regarding child development and parenting techniques as well as practical assistance such as linking families to services and social supports (Australian Institute of Family Studies, 2006). The 2021 Home Visiting Evidence of Effectiveness (HomVEE) assessed 65 early childhood home visiting models, of which 24 met the HHS criteria to prevent or reduce the incidence of child abuse and neglect (HHS, 2021).

For the present study, we are interested in formal home‐based intervention (Li, 2022; Van, 2020), and it is defined as receiving help from professional or paid workers rather than informal caregivers (i.e., most commonly family members and friends) in the home. Home is defined as an individual's place of residence (e.g., apartments and residential homes) rather than special institutions (e.g., hospice, long‐term care facilities, and nursing homes).

### 1.3 How the intervention might work

Home‐based child neglect prevention programs vary from country to country or from organization to organization (Ash, 2017; CDC, 2016; Curry, 2018; Guastaferro, 2022; HHS, 2018; UNICEF, 2023; Van, 2020). We will focus on all formal home‐based programs that provided support to families or individuals via the following groups (Donisch, 2022; HHS, 2020):

- Engagement of Family
  The intervention begins with engagement and trust building. The family will be helped to meet their own goals and their concerns are closely listened.
- Comprehensive Assessment of Child and Family
  To understand the child's health and development, the following information will be collected: the important relationships of the child with their parents as well as with other individuals who care for the child (e.g., early care providers); child trauma and other stressors (e.g., violence and separation); and the multiple challenges experienced by parents that interfere with their ability to protect, nurture, and support their child's development. Formal measures, conversations, observations, and records from other providers will also be included in the process.
- Development of Child and Family Plan of Care
  A family‐driven plan comprising comprehensive, well‐coordinated, therapeutic intervention goals, supports, and services will be developed in partnership with the parents or caregivers. This plan would reflect the parents' goals, priorities, strengths, culture, and needs.
- Parent–Child Psychotherapeutic Intervention
  The promotion of responsive nurturing through a parent–child psychotherapeutic approach will be designed to enhance the parent–child relationship as fundamental to the child's social–emotional health and cognitive development.
- Enhancement of Executive Functioning
  Self‐regulation and executive functioning capacity through psychotherapeutic intervention and the development and execution of the service plan, and caregiver mentoring will be promoted. Thus, they may be able to thoughtfully focus attention, plan, organize, solve problems, and succeed.
- Mental Health Consultation in Early Care and Education
  The mental health clinician would work with the early care and education environment to provide consultation to the teacher or caregiver. This is particularly critical when there are challenging behaviors within the classroom. The clinician would conduct observations, discuss the past and current behavior with the teacher, and help the teacher understand the meaning of the child's behavior. Together they would develop strategies that can meet the child's individual needs and coordinate efforts between early care and education and the child's home.
- Care Coordination
- The care coordinator would facilitate the coordination of services and the family's access to multiple resources throughout the community based on the collaborative planning with the child's parents. The care coordinator would listen carefully, always reflecting on the meaning of the service for the family. The care coordinator would also provide hands‐on assistance in obtaining information and collaborating with community providers, research program appropriateness and availability, and make and facilitate referrals to provider agencies.

### 1.4 Why it is important to do this review

As one of the most common types of child maltreatment, studies related to child neglect primarily focus on issues related to child maltreatment. Despite identifying potential interventions in these studies, there is a notable lack of dedicated descriptions for the population experiencing child neglect. Additionally, existing research indicates that current interventions mainly aim to reduce the recurrence of child maltreatment rather than preventing its initial occurrence. Further research is needed to investigate the effectiveness of family interventions and various associated factors in preventing child maltreatment. For instance, Mikton (2009) found that evidence for the effectiveness of four (home‐visiting, parent education, abusive head trauma prevention and multi‐component interventions) of the seven main types of interventions for preventing child maltreatment is promising. However, concerning home‐visiting, some studies included in the analysis consider the evidence inconclusive due to methodological issues, including surveillance bias. Euser (2015) synthesized findings from 27 independent samples from randomized controlled trials (RCTs) on the effectiveness of 20 different intervention programs aimed at preventing child maltreatment, and found currently existing programs appeared to only reduce and not prevent child maltreatment. Further moderator analyses revealed substantial variations in combined effects among different subsets of studies. Specifically, interventions with a moderate number of sessions (16–30) proved significantly more effective than those with fewer or more sessions. Significant effect sizes were observed only for interventions lasting 6–12 months, whereas interventions shorter than 6 months or longer than 12 months did not significantly reduce child maltreatment. The influence on various countries remains uncertain, and the type of sample emerged as a significant moderator affecting the overall effect size. Filene (2013); Segal (2012); Van der Put (2018) summarized findings on effects of interventions (including home visiting program) for preventing child maltreatment and by examining potential moderators and theory of this effect. Han (2022) investigated the effectiveness of home visiting programs targeting parents who have maltreated their children on the prevention of child maltreatment recurrence, the results showed that the risk of child maltreatment recurrence significantly decreased after a home visiting program was implemented.

In addition, some studies have assessed the evidence of only home‐based interventions for preventing and reducing child neglect. Further to the best of our knowledge, published systematic reviews of child neglect focused only on the discussion of disease risk factors and adverse consequences of child neglect, incidence and treatment of child neglect, description of evidence for child neglect home visiting programs, and effect of a specific family prevention model. At present, no systematic reviews have evaluated the effect of different home‐based intervention models to prevent child neglect. Mulder et al. found 24 potential risk domains of child neglect (Mulder, 2018), and Haslam and Taylor who reviewed the literature exploring the relationship between neglect and adolescent interpersonal functioning in peer relationships found that neglect, particularly emotional neglect, is associated with reduced relationship quality and that there is consistent evidence that neglect increases the risk of gang involvement and deviant peer affiliation (Haslam, 2022). An article (Stoltenborgh, 2013) published in 2013 performed a meta‐analysis of the prevalence of physical and emotional neglect but failed to evaluate the effectiveness of interventions for child neglect and the specific content of these interventions. Allin et al. identified 14 studies published from 1985 to 2002 and evaluated the available evidence regarding the effectiveness of child neglect treatment programs (Allin, 2005). Peacock et al. systematically reviewed the effectiveness of home‐visiting programs on the developmental and health outcomes of young children belonging to disadvantaged families, including studies that assessed child abuse and neglect (Peacock, 2013). In 2018, the USPSTF reviewed evidence that included home visitation‐based prevention interventions to prevent child maltreatment. Although insufficient evidence was found to assess the benefits and harms of preventing maltreatment among children without the signs or symptoms of maltreatment, this recommendation did not assess the effectiveness of home visitation programs for other outcomes (e.g., improving child and maternal health, encouraging positive parenting, or promoting child development) (Curry, 2018; Viswanathan, 2018). Bilukha et al. assessed the effectiveness of home‐visiting programs in preventing violence (Bilukha, 2005), whereas McGinn et al. assessed the effectiveness of the formal use of family group decision‐making regarding child safety, permanence, child and family well‐being, and client satisfaction with the decision‐making process (McGinn, 2020). In addition, some reviews integrated the evidence of the effectiveness of other behavioral interventions (such as family group decision‐making and those implemented in schools or healthcare facilities) to reduce and prevent child neglect and related health outcomes (McGinn, 2020; Shelley, 2013; Yildiz, 2018).

## 2 OBJECTIVES

The objectives of the present study are to answer the following questions:

- What types of home‐based interventions are currently being studied to prevent child neglect?
- How effective are the different home‐based interventions for preventing child neglect?
- What are the causes of heterogeneity among included studies and their impact on study effects?

## 3 METHODS

### 3.1 Criteria for considering studies for this review

#### 3.1.1 Types of studies

The review procedure will conform to the Cochrane Collaboration's Effective Practice and Organization of Care (EPOC) criteria for study selection (EPOC, 2017). We will include individual and cluster RCTs, nonrandomized controlled trials (NRCTs), and controlled before and after (CBA) studies as well as interrupted time series or repeated time measures studies (ITSs). However, cross‐sectional studies will be excluded. Moreover, studies that do not include a control group will be excluded from the review as it is difficult to attribute causation in a study design without a control group (Dewidar, 2021).

#### 3.1.2 Types of participants

This review will primarily focus on research results on the primary prevention of child neglect rather than on preventing recidivism.

The target population will include the general population (aged 0–18 years) and their parents, guardians, or other informal caregivers in families identified as at‐risk for child neglect. Families at risk may exhibit factors such as food insecurity, financial stress, intimate partner violence, social isolation, mental health issues, or a substance use disorder (Donisch, 2022).

#### 3.1.3 Types of interventions

This review will include any type of formal home‐based programs designed to emphasize primary prevention of child neglect (Van, 2020). The interventions provided by paid professionals and teams in families that are deemed at‐risk but not currently experiencing neglect. Exclusions will be made for interventions provided by informal caregivers, families, and friends. Studies conducted in special institutions (e.g., hospitals or nursing homes) or through telemedicine or telecare will also be excluded.

#### 3.1.4 Types of outcome measures

The development of outcomes would be mainly based on HomVEE reviews (HHS, 2021). A study measuring the effectiveness of home‐based intervention on at least one or more of the following outcomes will be included in this study.

##### Primary outcomes

- Occurrence of child neglect (as measured by the official records of child self‐reports to the program staff, child protective services, or police);
- Positive parenting practices that include the knowledge of child development, safety practices, supportive behavior, and engagement with the child; promotion of learning and child development; disciplinary practices; and general parenting practices such as bedtime routines (outcomes in this domain include the observational measures of parent–child interactions or the home environment and parent self‐reports on parenting attitudes and practices);
- Reductions in juvenile delinquency, family violence, and crime (outcome measures in this domain include the incidence of parent and youth antisocial behavior based on archived data from state records as well as from the parent, teacher, and youth self‐reports on antisocial behaviors).

##### Secondary outcomes

- Child development and school readiness that include the child's social behaviors, attachment to a parent or caregiver, social–emotional or psychological development, or cognitive and academic development (outcome measures in this domain include direct child assessments, reviews of school records, direct observations of children's behavior, and parent and teacher reports on standardized measures);
- Family economic self‐sufficiency (outcome measures in this domain include the measures of public assistance receipts that are based on government administrative records and self‐reports on service receipts and economic outcomes);
- Linkages and referrals (outcome measures in this domain include the reviews of home visitor, medical, or school records for indications that the child or family had received a referral to other services in the community as well as parent reports on receiving a referral and being aware of other services in the community).

### 3.2 Search methods for identification of studies

According to the Campbell Searching for Studies Guide (Kugley, 2016), the pilot searches have been conducted, we will conduct a systematic search of all retrievable studies and reports, as well as hand‐search journals to determine the best available evidence. We will complement our searches using Google and Google Scholar to facilitate access to technical reports or governmental publications. Additionally, we plan to conduct forward citation searching with Google Scholar to identify studies citing our included studies. Furthermore, we will perform backward citation searching for references cited in both primary studies and reviews related to the topic identified during our current search. Simultaneously, the search strategy will be determined based on the listed search terms, with no restrictions on date, language, or location.

#### 3.2.1 Electronic searches

The Cochrane Database of Systematic Reviews and the Central Register of Controlled Trials (https://www.cochranelibrary.com/).

The Campbell Library (https://www.campbellcollaboration.org/).

Medline (EBSCO).

Scopus (Elsevier).

PubMed (excluding MEDLINE).

Embase (https://www.embase.com/).

Web of Science Core Collection (Web of Science).

APA PsycINFO (EBSCO).

CINAHL (EBSCOhost).

China National Knowledge Infrastructure (https://www.cnki.net/).

VIP Chinese Science and Technique Journals Database (http://www.cqvip.com/).

The Chinese Biomedical Database (SinoMed).

Wanfang Data (https://www.wanfangdata.com.cn).

##### Search terms and key words

Below is a list of key words that will be used to search electronic databases and agency websites. The search terms necessarily must be adapted for each database, although the concepts of child neglect, and home remain constant. For example, the PubMed search strategy is shown in the Table 1.

**Table 1:** PubMed search strategy.

#	Searches
**#1**	“Child”[Mesh] OR “Adolescent”[Mesh] OR “Infant”[Mesh]
**#2**	baby[Title/Abstract] OR babies[Title/Abstract] OR infant*[Title/Abstract] OR child*[Title/Abstract] OR preschool*[Title/Abstract] OR pre‐school*[Title/Abstract] OR teen*[Title/Abstract] OR adolescen*[Title/Abstract] OR youth[Title/Abstract] OR pediatric[Title/Abstract] OR paediatric[Title/Abstract] OR toddler[Title/Abstract] OR minors[Title/Abstract] OR offspring[Title/Abstract] OR juvenile*[Title/Abstract] OR junior*[Title/Abstract] OR Little one[Title/Abstract] OR tot[Title/Abstract]
**#3**	#1 OR #2
**#4**	abuse*[Title/Abstract] OR maltreat*[Title/Abstract] OR mal‐treat*[Title/Abstract] OR mistreat*[Title/Abstract] OR neglect*[Title/Abstract]
**#5**	#3 AND #4
**#6**	“Child Abuse”[Mesh]
**#7**	#5 OR #6
**#8**	“Home Health Nursing”[Mesh] OR “Home Care Agencies”[Mesh] OR “Home Nursing”[Mesh] OR “Home Care Services”[Mesh] OR Community Health Nursing[Mesh] OR Social Support[Mesh] OR house calls[Mesh]
**#9**	home‐based OR home based OR family‐based OR family based
**#10**	home[Title/Abstract] OR in‐home[Title/Abstract] OR at‐home[Title/Abstract] OR house[Title/Abstract] OR community[Title/Abstract] OR neighbo? rhood[Title/Abstract] OR family[Title/Abstract] OR lay[Title/Abstract] OR volition[Title/Abstract] OR domiciliary[Title/Abstract] OR respite[Title/Abstract] OR residential[Title/Abstract] OR apartment[Title/Abstract]
**#11**	care*[Title/Abstract] OR service*[Title/Abstract] OR sta*[Title/Abstract] OR worker*[Title/Abstract] OR nurs*[Title/Abstract] OR health personnel[Title/Abstract] OR pharmacist[Title/Abstract] OR occupational[Title/Abstract] OR health care[Title/Abstract] OR patient care team*[Title/Abstract] OR patient care agenc*[Title/Abstract] OR patient care institute*[Title/Abstract] OR patient care compan*[Title/Abstract] OR visit[Title/Abstract] OR support[Title/Abstract] OR program*[Title/Abstract] OR prevent*[Title/Abstract] OR involve*[Title/Abstract] OR interfere*[Title/Abstract] OR mediat*[Title/Abstract] OR interce*[Title/Abstract] OR interven*[Title/Abstract] OR meddl*[Title/Abstract] OR interpos*[Title/Abstract] OR assist*[Title/Abstract] OR arbitrat*[Title/Abstract] OR treat*[Title/Abstract] OR action[Title/Abstract] OR influence[Title/Abstract] OR help[Title/Abstract] OR aid[Title/Abstract]
**#12**	#10 AND #11
**#13**	#8 OR #9 OR #12
**#14**	#7 AND #13

###### Child neglect

baby OR babies OR infant* OR child* OR preschool* OR pre‐school* OR teen* OR adolescen* OR youth OR pediatric OR paediatric OR toddler OR minors OR offspring OR juvenile* OR junior* OR Little one OR tot

AND

abuse* OR maltreat* OR mal‐treat* OR mistreat* OR neglect*

###### Home‐based interventions

home OR in‐home OR at‐home OR house OR community OR neighbo? rhood OR family OR lay OR volition OR domiciliary OR respite OR residential OR apartment

AND

care* OR service* OR sta* OR worker* OR nurs* OR health personnel OR pharmacist OR occupational OR health care OR patient care team* OR patient care agenc* OR patient care institute* OR patient care compan* OR visit OR support OR program* OR prevent* OR involve* OR interfere* OR mediat* OR interce* OR interven* OR meddl* OR interpos* OR assist* OR arbitrat* OR treat* OR action OR influence OR help OR aid

OR

home‐based OR home based OR family‐based OR family based

#### 3.2.2 Searching for other resources

ClinicalTrials.gov (clinicaltrials.gov).

Grey Literature Report in 1999 to 2016 (www.greylit.org).

The International Clinical Trials Registry Platform of the World Health Organization (ICTRP, apps.who.int/trialsearch/Default.aspx).

ProQuest Dissertations & Theses Global (ProQuest).

Google (https://www.google.co.uk/).

Google Scholar (scholar.google.be).

Social Care Institute for Excellence (https://www.scie.org.uk/).

The Latin American and Caribbean Centre (https://www.lse.ac.uk/lacc).

The American Institutes for Research (https://www.air.org/).

The California Evidence‐Based Clearinghouse for Child Welfare (CEBC) (https://www.cebc4cw.org).

Child First (https://www.childfirst.org/).

NSPCC Learning (https://learning.nspcc.org.uk/).

UK HealthCare (https://ukhealthcare.uky.edu/).

Australian Institute of Family Studies (https://aifs.gov.au/).

U.S. Department of Health & Human Services (https://www.hhs.gov/).

Canadian Child Welfare Research Portal (https://cwrp.ca/child-abuse-neglect).

International Society for Prevention of Child Abuse and Neglect (www.ispcan.org/).

Promising Practices Network operated by the RAND Corporation (http://www.promisingpractices.net/).

National Resource Centre for Community‐Based Child Abuse Prevention (CBCAPP) (http://friendsnrc.org/).

Coalition for Evidence‐Based Policy (http://coalition4evidence.org/).

Institute of Education Sciences What Works Clearinghouse (http://ies.ed.gov/ncee/wwc/).

##### In addition, to ensure we identify the most recent references, we will hand‐search the last 5 years of key journals such as

Child Abuse & Neglect: The International Journal (https://www.ispcan.org/learn/can-international-journal/).

Child Welfare (https://www.cwla.org/child-welfare-journal/).

Children and Youth Services Review (https://www.sciencedirect.com/journal/children-and-youth-services-review).

Social Service Review (https://www.journals.uchicago.edu/toc/ssr/current).

Child Maltreatment (https://journals.sagepub.com/home/CMX).

Journal of Social Services Research (https://www.tandfonline.com/journals/wssr20).

Social Work (https://academic.oup.com/sw).

Research on Social Work Practice (https://journals.sagepub.com/home/rsw).

Social Work Research (https://academic.oup.com/swr).

Child Abuse Review (https://onlinelibrary.wiley.com/journal/10990852).

Prevention Science (https://www.springer.com/journal/11121).

Journal of Primary Prevention (https://journals.scholarsportal.info/browse/0278095x/v14i0004).

### 3.3 Data collection and analysis

#### 3.3.1 Selection of studies

**Figure 1:**
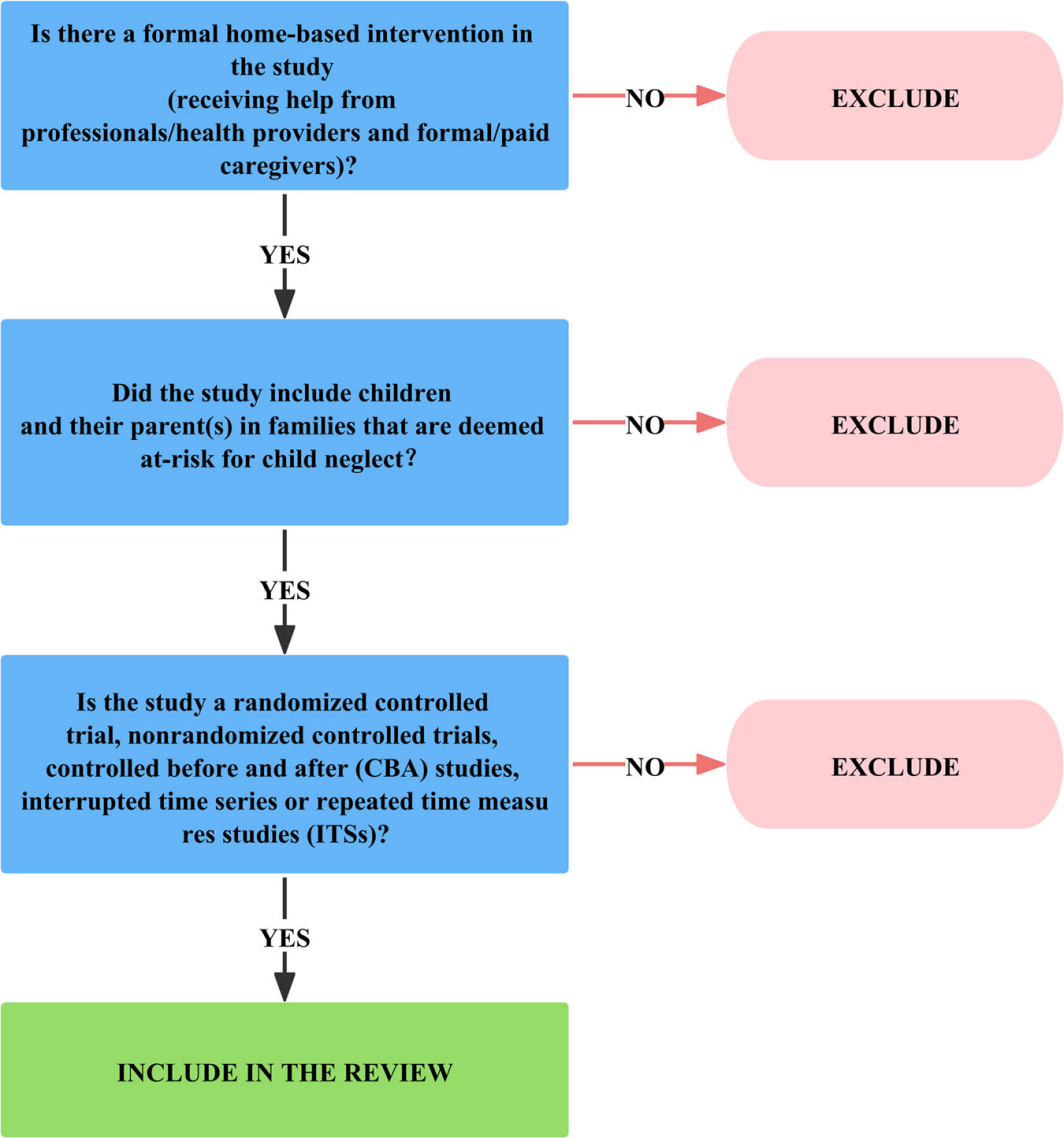
Screening tool.

Two authors will independently perform a rigorous study screening (Figure 1). The entire screening process will be executed using the EndNote X9 software and Rayyan QCRI. First, duplications will be removed using two software and then the two authors will perform preliminary screening based on the study title and abstract. Second, for the initially included studies, the two authors will rescreen them by searching their full text to determine the final list of studies to be included. During this process, disagreements between the two reviewers, if any, will be resolved by consultation with a third author. At the same time, during the preliminary screening, if there is insufficient information to exclude a study, the study will be further screened using its full text.

#### 3.3.2 Data extraction and management

Two authors will independently extract data based on the predesigned tables of this review using an Excel spreadsheet. Quantitative data will be entered into the RevMan version 5.3 software and evaluated for accuracy. The extracted content would mainly include the following: the first author name, publication year, country, education level (parents), family income, sex, and age characteristics of participants; sample size; study design; statistical analysis; intervention; and other data. The following intervention (and comparison interventions) data will be extracted wherever possible based on established domains (Campbell, 2018): the name and type of intervention; the logic, mechanisms, or rationale of intervention; intervention materials; intervention goals; breadth of services spanned; roles and range of tasks; provider/delivery method of intervention and intervention settings; intensity and duration of intervention; intervention adaptation (adaptation during implementation to respond to changing circumstances); intervention integrity/fidelity (degree to which the intervention was delivered according to the original design); and any contextual factors that may shape implementation effectiveness (Supporting information: Appendix 1).

#### 3.3.3 Assessment of risk of bias in included studies

The risk of bias in the included RCTs will be assessed using the tool recommended by the Cochrane Handbook Version 5.1.0 (Cochrane Collaboration; United Kingdom) as per the following seven aspects: random sequence generation, allocation concealment, participant and personnel blinding, outcome assessment blinding, incomplete outcome data, selective reporting, and other bias. Every item will be classified as yes (“low risk of bias”), no (“high risk of bias”), or unclear (“moderate risk of bias”). When the risk of bias of all seven components is defined “low risk of bias,” the study will be overall considered “low risk of bias.” At the same time, when one or more of the seven bias components is classified high risk, the study will be graded as “high risk of bias.” In other cases, the study will be graded “unclear risk.” Disagreements in bias classification will be resolved by discussions between the two reviewers and, if necessary, through discussions with the other authors.

The modified EPOC risk of bias tool will be employed to evaluate the quality of ITS and CBA studies (EPOC, 2017), and the validated revised methodological index for nonrandomized studies (MINORS) criteria will be used to assess the risk of bias in NRCTs (Slim, 2003).

#### 3.3.4 Measures of treatment effect

For continuous outcomes, we will calculate standardized mean difference (SMD) or Cohen's d as the main metric for such outcomes. In the event of a small sample size, SMD will be corrected by transforming the point estimate into Hedges' g, using the formula in Lipsey (2001). The estimated parameters will be reported along with 95% confidence intervals (CIs). In two‐group pre‐post experimental studies, we will extract the means and standard deviations (SDs) before and after the intervention. We will apply the difference‐in‐difference adjustment, subtracting the pretest mean from the posttest mean, and subsequently calculate the corresponding SDs for both groups (Higgins, 2023). If SDs are not available, we will calculate them using *p* values, *t* values, or CIs (if reported) using the Campbell Collaboration effect size calculator (https://www.campbellcollaboration.org/escalc/html/EffectSizeCalculator-SMD-main.php). If dichotomous outcomes are reported, we will calculate risk ratios (RRs) with 95% CIs.

If different outcome types exist under the same outcome construct, for comparability of estimated effect sizes, we will we convert estimates to the most common standardized metric (Higgins, 2023). We will run sensitivity analysis for any differences caused by potential transformation.

We plan to extract as many relevant effect sizes as possible from included primary studies. This may be where studies report the effect on multiple outcomes or for multiple waves of data collection. Thus, we will include dependent effect sizes in our meta‐analysis. It is likely we will have both between‐study (e.g., same research teams conducting multiple evaluations) and within‐study (e.g., multiple follow‐ups from same study sample) dependency in the meta‐analysis. This dependence structure will inform our chosen meta‐analytical model, and consider the use of robust variance estimation (Pustejovsky, 2021).

#### 3.3.5 Unit of analysis issues

We recognize that some included studies may have evaluated one or more interventions together. In such cases, we will include each pairwise comparison separately. In cases of multiple intervention durations, we will analyze each outcome at each intervention duration separately. Comparable studies taking measures at an intervention duration will be analyzed together and grouped as follows: short term (<6 months), medium term (6 months to <12 months), and long‐term (≥12 months). For trials with more than two arms, we will split the “shared” group into two or more groups with smaller sample sizes and include two or more (reasonably independent) comparisons, as described in the Cochrane Handbook (Higgins, 2023). For cluster randomized trials where groups of people are allocated to interventions, we will assess these studies for unit of analysis errors (Welch, 2018). If unit of analysis errors (i.e., analysis at the level of the individual, without adjusting for clustering) exist, we will inflate the SD using the variance inflation factor for each intervention arm using an intra‐cluster correlation coefficient (ICC) from a similar trial or from a database of ICCs. For dichotomous outcomes, we will use methods suggested in the Cochrane Handbook for Systematic Reviews of Interventions to adjust the numerator and denominator for unit of analysis errors (Higgins, 2023).

#### 3.3.6 Dealing with missing data

We will attempt to contact the study authors to supplement any missing or unreported data. If data cannot be obtained, we will not include the study in the meta‐analysis and the extent to which the results or conclusions of the review might be affected by this exclusion will be assessed and discussed.

#### 3.3.7 Assessment of heterogeneity

Forest plots will be constructed to visually investigate overlaps in the CIs for the results of the individual studies. In addition, Dixon's *Q*‐test and the *I*‐squared (*I* ^2^) statistical tests will be used to assess heterogeneity in the results. If the *p*‐value is <0.05 and *I* ^2^ is >50%, the result will be recognized as heterogeneous, and subgroup and sensitivity analyses will be performed to explore the possible reasons for heterogeneity.

We will also use random effects meta‐regression to investigate the association between moderator variables and heterogeneity of treatment effects (Higgins, 2023). If these strategies are not possible (e.g., if we do not have sufficient number of studies or data), we will discuss and explore the factors which may be driving the heterogeneity of results narratively (Villar, 2023).

#### 3.3.8 Assessment of reporting biases

We will establish funnel plots and visually examine the signs of asymmetry to investigate publication bias and subsequently use Egger's test as a formal test of publication bias when the number of the included studies on an outcome is ≥10 (*n* ≥ 10).

#### 3.3.9 Data synthesis

The meta‐analysis will be performed using the RevMan version 5.3 software. We will pool results from clinically similar interventions. For dichotomous outcomes, the Mantel–Haenszel method will be used, and RRs will be combined with 95% CIs from the included studies. For continuous outcomes, the inverse‐variance method will be used, and MD, or SMD (if studies measure the outcome on different assessment scales), along with 95% CIs will be calculated. Because heterogeneity exists in theory due to the variety of interventions and contexts that could be included in the review, we will use random‐effects meta‐analysis. Subgroup analysis will be performed to examine the effects of varying interventions and populations as well as the heterogeneity of included studies. In case a quantitative synthesis is not possible, the study findings will be discussed narratively (Campbell, 2020; Yang, 2018a, 2018b). The data (e.g., events, sample size, and point estimates) will be presented in the form of a forest plot or table and grouped as per different outcomes and comparisons. For each comparison and outcome, a description of the review findings would be provided, thereby clarifying the direction of outcome of each study (Campbell, 2020).

#### 3.3.10 Subgroup analysis and investigation of heterogeneity

If sufficient data are available, subgroup analysis will be performed to assess effects and explore the potential sources of heterogeneity in terms of the following factors:

- Home‐based intervention: variations in the type of intervention; intervention goals; intervention materials; provider/delivery method of intervention; intensity of intervention; duration (grouped as short term “<6 months,” medium term “6 months to <12 months,” and long term “≥12 months”); intervention settings; the logic, mechanisms, or rationale of intervention; breadth of services spanned; and roles and range of tasks will be included wherever possible.
- Population: variations in age, sex, education level (parents), countries, family income, and others will be included (if possible).
- Study design and the risk of bias of included studies: we will restrict subgroup analyses to outcomes that have at least two studies available. The subgroups will be compared using the formal statistical test for subgroup differences in RevMan 5.3, and the results will be interpreted with caution.

#### 3.3.11 Sensitivity analysis

Sensitivity analysis will be performed to determine whether the pooled effect sizes are stable across the components of risk of bias by limiting the meta‐analysis to a subset of all studies included in the original meta‐analysis. For each domain of the risk of bias checklists, sensitivity will be considered, and the analysis will be limited to studies with low RoB. In addition, the “leave‐one‐study‐out” method sensitivity analysis will be used to evaluate how each study affects the effect size estimates (Higgins, 2023). It will be also necessary to run sensitivity analysis on the statistical procedures to compute effect sizes (e.g., transforming effect sizes), and the inclusion of reports presenting missing/incomplete data, among others.

## SOURCES OF SUPPORT

**Internal sources**

- No sources of support provided

**External sources**

- No sources of support provided

## CONTRIBUTIONS OF AUTHORS

- Content: Yanfei Li, Rui Li, Meixuan Li
- Systematic review methods: Xiuxia Li, Kehu Yang
- Statistical analysis: Meixuan Li, Yanfei Li, Zhitong Bing
- Information retrieval: Yanfei Li, Rui Li, Meixuan Li, Xiuxia Li, Kehu Yang, Zhitong Bing
- Information specialist: Kehu Yang, Zhitong Bing

## DECLARATIONS OF INTEREST

All authors have no conflicts of interest

## Supporting information

Supporting information.
