# Supplementary material for: PROTOCOL: Effectiveness of home‐based interventions to prevent child neglect: A systematic review
Source: Campbell Syst Rev. 2023 Dec 6;19(4):e1373. doi: 10.1002/cl2.1373 (PMC10698703; doi:10.1002/cl2.1373)
Supplement: Supplementary file 1 — Supporting information. [file CL2-19-e1373-s001.docx]

Appendices

## 1 Article coding and data extraction

| Publication details | Author (S) |  |
| --- | --- | --- |
|  | Year of publication |  |
|  | Country |  |
| Population | Age (mean, SD, range, as reported) |  |
|  | Education level (parents) |  |
|  | Family income |  |
|  | Sex/Gender | 0-25% female included |
|  |  | 25-50% female included |
|  |  | 50-75% female included |
|  |  | 75-100% female included |
| WHO Regions | South-East Asia |  |
|  | Western Pacific |  |
|  | European |  |
|  | African |  |
|  | The Americas |  |
|  | Eastern Mediterranean |  |
| World Bank Classification by Income | Low-income economies |  |
|  | Lower-middle income economies |  |
|  | Upper-middle income economies |  |
|  | High-income economies |  |
| Study design | RCTs |  |
|  | NRCTs |  |
|  | CBA |  |
|  | ITSs |  |
| Intervention | The name and type of intervention |  |
|  | The logic, mechanisms, or rationale of intervention |  |
|  | Intervention materials |  |
|  | Intervention goals |  |
|  | The breadth of services spanned |  |
|  | The roles and range of tasks |  |
|  | The provider/delivery method of intervention |  |
|  | Intervention settings |  |
|  | Intervention adaptation (adaptation during implementation to respond to changing circumstances) |  |
|  | Intervention integrity/fidelity (degree to which the intervention was delivered according to original design) |  |
|  | Other (any contextual factors that may shape implementation effectiveness) |  |
|  | Duration of intervention | Short‐term (less than 6 months) |
|  |  | Medium‐term (6 months to less than 12 months) |
|  |  | Long‐term (12 months or more) |
|  | Number of sessions per week |  |
|  | Number of sessions per day |  |
|  | Duration of each session |  |
|  | Time between sessions |  |
| Comparison/Control | Usual Care |  |
|  | No intervention |  |
|  | Other |  |
| Simple size | The number of participants in intervention group/control group |  |
| Outcomes | Occurrence of child neglect |  |
|  | Positive parenting practices |  |
|  | Reductions in juvenile delinquency, family violence, and crime |  |
|  | Child development and school readiness |  |
|  | Family economic self-sufficiency |  |
|  | Linkages and referrals |  |
| Quality assessment | High risk of bias |  |
|  | Unclear risk of bias |  |
|  | Low risk of bias |  |
| Data for analysis | Quantitative information on per outcome (e.g., means, standard deviations, t‐values, simple size) (Note: all related outcomes will be extracted from the study and will be recorded in an Excel file for effect size calculations) | |
